# Identification of Human Disease Genes from Interactome Network Using Graphlet Interaction

**DOI:** 10.1371/journal.pone.0086142

**Published:** 2014-01-22

**Authors:** Xiao-Dong Wang, Jia-Liang Huang, Lun Yang, Dong-Qing Wei, Ying-Xin Qi, Zong-Lai Jiang

**Affiliations:** 1 Institute of Mechanobiology and Medical Engineering, School of Life Sciences & Biotechnology, Shanghai Jiao Tong University, Shanghai, China; 2 Bioinformatics, Integrated Platform Science, GlaxoSmithKline Research and Development China, Shanghai, China; 3 Bio-X Institutes, Key Laboratory for the Genetics of Developmental and Neuropsychiatric Disorders, Shanghai Jiao Tong University, Shanghai, China; 4 State Key Laboratory of Microbial Metabolism, School of Life Sciences & Biotechnology, Shanghai Jiao Tong University, Shanghai, China; Huazhong University of Science and Technology, China

## Abstract

Identifying genes related to human diseases, such as cancer and cardiovascular disease, etc., is an important task in biomedical research because of its applications in disease diagnosis and treatment. Interactome networks, especially protein-protein interaction networks, had been used to disease genes identification based on the hypothesis that strong candidate genes tend to closely relate to each other in some kinds of measure on the network. We proposed a new measure to analyze the relationship between network nodes which was called graphlet interaction. The graphlet interaction contained 28 different isomers. The results showed that the numbers of the graphlet interaction isomers between disease genes in interactome networks were significantly larger than random picked genes, while graphlet signatures were not. Then, we designed a new type of score, based on the network properties, to identify disease genes using graphlet interaction. The genes with higher scores were more likely to be disease genes, and all candidate genes were ranked according to their scores. Then the approach was evaluated by leave-one-out cross-validation. The precision of the current approach achieved 90% at about 10% recall, which was apparently higher than the previous three predominant algorithms, random walk, Endeavour and neighborhood based method. Finally, the approach was applied to predict new disease genes related to 4 common diseases, most of which were identified by other independent experimental researches. In conclusion, we demonstrate that the graphlet interaction is an effective tool to analyze the network properties of disease genes, and the scores calculated by graphlet interaction is more precise in identifying disease genes.

## Introduction

Identifying human disease genes is an important task in biomedical researches. Besides the experimental and clinical approaches which identify individual disease genes directly, there are a growing number of methods to predict more disease genes by computational approaches [1]. Most of these studies are based on the disease gene databases, such as Online Mendelian Inheritance in Man (OMIM) [2], which is used to disease gene identification, human disease network construction, and et al [3].

Interactome networks [4], especially protein-protein interaction (PPI) network have been used in many areas, e.g. protein complex detection [5], [6], protein function prediction [7], signaling pathway extraction [8], disease diagnosis [9], disease comorbidity analysis [10], and essential gene identification [11]. In recent years, several approaches are designed to predict human disease genes according to their relationship with known disease genes by using the interactome networks [12]. The hypothesis of these methods is that if a candidate gene has close relationship with known disease genes in the network under some measure, it is considered as a disease gene as well.

The simplest method to identify disease genes is based on the neighborhood. The gene, which directly links with at least 1 known disease gene in a network will be identified as a disease gene. To improve the precision, Oti, et al. limited the genes by checking whether their chromosomal regions located within one or more disease loci [13]. Furthermore, if limited the genes to which linked with at least 2 and 3, respectively, known disease genes, the precision increased, but the recall decreased [14]. Researchers developed new criteria in order to identify more disease genes while keeping high precision. Lage, et al. designed Bayesian predictor to identify disease genes from protein complexes, and provided novel candidate genes implicated in disorders such as retinitis pigmentosa, epithelial ovarian cancer, and et al [15]. However, the calculation is time-consuming, and the precision is not high enough (less than 0.65). Xu, et al. combined neighborhood and network topological characteristics by k-nearest neighbors (KNN) algorithm to classify the disease genes from other genes [16], which improved the precision to about 0.75. CIPHER, a regression based algorithm, also increased the precision [17]. Kohler, et al. [18] adopted random walk algorithm to identify disease genes from 5 species of PPI networks. It is more convenient to calculate, and is better than other network-based methods with the precision more than 0.9 [14]. However, when the number of identified genes increases, the precision of random walk decreases rapidly. Other methods integrated multiple heterogeneous data sources to improve the performance, such as Endeavour [19], MGC [20], and functional linkage network (FLN) based approach [21]. However, it is still necessary to find better disease gene identification method to identify more disease genes conveniently, and get high precision at the same time.

To identify disease genes precisely and conveniently, we proposed a new approach based on graphlet. Graphlet is an effective tool to analyze network properties. It had been applied to compare networks by calculating the graphlet degree distribution of each network [22]. The vector of graphlet degree is called graphlet signature. The elements of the graphlet signature indicate the amount of different graphlet automorphism orbits. Graphlet signature had been used to uncover network functions [23] and analyze protein properties in networks [24], [25]. Genes with similar graphlet signature in the network may have similar functions [23]–[25]. In our present research, we found that graphlet could be considered as a new linkage type between two nodes in a network. Two nodes in the same graphlet are considered to interact with each other even though there is no direct linkage between them. Thus, the linkage can be redefined, and we called the new linkage type as graphlet interaction.

In this paper, we developed a new approach to identify human disease genes using graphlet interaction. Firstly, graphlet interactions between random picked gene pairs in the disease loci and the known disease gene pairs of same disease families in OMIM were calculated, respectively. It revealed that the graphlet interaction between disease genes was significant different from that between random picked genes. Then, candidate genes were ranked according to the scores which were calculated by their graphlet interaction with known disease genes. The precision was evaluated using leave-one-out cross-validation compared with other approaches. Finally, new disease genes of 4 common diseases, i.e. breast cancer, colorectal cancer, prostate cancer and diabetes, were predicted and analyzed.

## Methods

### 2.1 Graphlet and graphlet interaction

**Figure 1:**
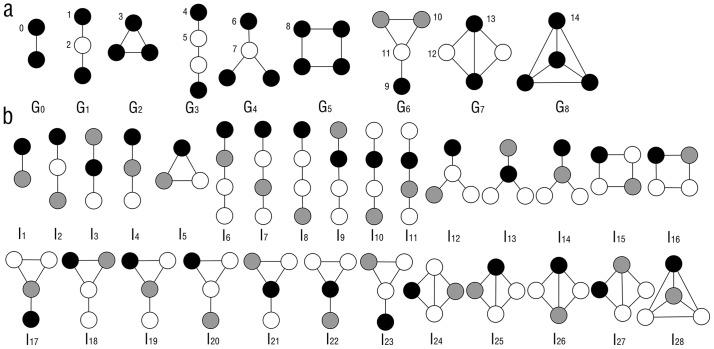
Graphlet and graphlet interaction isomers. The figure showed the introduction of graphlet and graphlet interaction. **a**. Graphlet types which were labelled by *G_0_* to *G_8_*, and automorphism orbits which were labelled by number 0 to 14. Black, white and gray nodes represented different orbits in the same graphlet. **b**. Graphlet interaction isomers *I_1_* to *I_28_* between two nodes which were marked with black and gray.

The graphlet is a type of small connected subgraph which is non-isomorphic [22]. A whole large network is consisted of the graphlets. Different network has different number of graphlets. Computing all the graphlets of a network is a NP-complete problem. In this paper, only graphlets with not more than 4 nodes were considered. The graphlets are shown in Figure 1a. There are 9 types of graphlets labeled with *G_0_* to *G_8_* with 2, 3 or 4 nodes, 1 graphlet (*G_0_*) with 2 nodes, 2 graphlets (*G_1_, G_2_*) with 3 nodes and 6 graphlets (*G_3_–G_8_*) with 4 nodes. Nodes in the graphlets occupy different positions, which are called automorphism orbits [22]. Nodes in the same automorphism orbits have the same local topological properties in the graphlet. These 9 types of graphlets have 15 automorphism orbits (Figure 1a). More detailed information about graphlet is described in the previous publications [22]–[25].

Graphlet interaction describes the relationship between 2 nodes. There is a graphlet interaction between the two nodes in the same graphlet. It was defined by Equation (1). There is a graphlet interaction between node *i* and node *j* of graph *H* when satisfy
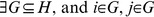
(1)where *G* is a graphlet in *H,* and *V*(*G*) is the nodes set of *G*.

In Figure 1b, the black and gray nodes represent the nodes *i* and *j* which have a graphlet interaction. Thus, there are different types of relationships between the two nodes (black and gray) according to their different automorphism orbits. The different types of relationships between the 2 nodes are called graphlet interaction isomers. For example, the graphlet interaction isomer *I_2_*, *I_3_* and *I_4_* are all similar as the graphlet *G_1_*. However, the nodes *i* and *j* (black and gray) are in different automorphism orbits of graphlet *G_1_*, which should be seen as different graphlet interaction isomers. The graphlet interaction is a vector, of which every element represents the number of the corresponding graphlet interaction isomers. Since computation of all types of graphlet interaction isomers in a network is an NP-complete problem, only not more than 4 nodes graphlets were considered. There are 28 graphlet interaction isomers labeled as *I_1_* to *I_28_* (Figure 1b). The graphlet interaction vector has 28 elements corresponding to the 28 types of graphlet interaction isomers.

### 2.2 Computation of graphlet interaction

The graph *H* is represented by the adjacency matrix *A*  =  (*a_ij_*). If there is an edge between nodes *i* and *j* of *H*, *a_ij_*  = 1; otherwise, *a_ij_*  = 0. When counting the graphlet interaction between nodes *i* and *j*, the number of isomer *I_k_* was calculated by the equation
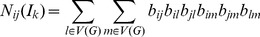
(2)
*b* is a variable to make equation (2) clear and calculated by the following



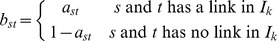
(3)In the above equations, *N* *_ij_*(*I_k_*) represents the number of the isomer *I_k_* between nodes *i* and *j*, *l* and *m* represent the other 2 nodes besides nodes *i* and *j*, and *a_ij_* represents the elements of adjacency matrix *A*. *i*, *j*, *l* and *m* are all unequal. When the nodes *i*, *j*, *l* and *m* in the network constitute a graphlet interaction isomer, all the 6 items, i.e. *b_ij_*, *b_il_*, *b_jl_*, *b_im_*, *b_jm_*, *b_lm_*, are equal to 1. The product will be 1, and added to the number of the corresponding isomer. After all the nodes being traversed, the total number of the isomer from node *i* to *j* can be calculated. The larger number of the isomers *I_k_* suggests the closer relationship between the two nodes *i* and *j*.

The computing based on Equation (2) is too time-consuming. Hence, in practice the isomers were counted by the vectors of the adjacency matrix like *a_i_* and *a_j_*. For example, the number of isomer *I_2_* was computed by *N* *_ij_*(*I_2_*)  =  *a_i_* * *a_j_*, where *N* *_ij_*(*I_2_*) means the number of *I_2_* between node *i* and *j* and * means inner product of two vectors.

The graphlet interaction has directions, which represents that if calculating the graphlet interaction of two nodes, *i* and *j*, the graphlet interaction from node *i* to node *j* does not equal to that from *j* to *i*. There are some symmetrical graphlet isomers, such as *I_3_* and *I_4_*. *N* *_ij_*(*I_3_*)  = *N* *_ij_*(*I_4_*), which means that the third element of graphlet interaction vector from *i* to *j* is equal to the fourth element of that from *j* to *i*.

### 2.3 Ranking candidate genes by graphlet interaction scores

In order to identify disease genes by using graphlet interaction, the candidate genes were ranked by the scores based on graphlet interaction. A gene with a higher score may have closer relation with known disease genes, and thus have higher probability to be a disease gene as well. The graphlet interaction scores were calculated by the following equation
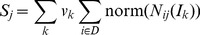
(4)where *S_j_* means the score of the gene *j*, *v_k_* is the weight of the *k*th isomer, *D* is the known disease gene set belong to some disease family, norm(*N* *_ij_*(*I_k_*)) is the normalized graphlet interaction, which was calculated by
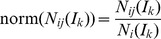
(5)where *N* *_ij_*(*I_k_*) is the number of the graphlet interaction isomer *I*_k_ from known disease gene *i* to candidate gene *j*, which is calculated by Equation (2). *N* *_i_*(*I_k_*) represents the total number of graphlet isomer *I*_k_ from known disease gene *i* to other genes. The *N* *_i_*(*I_k_*) was calculated as
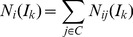
(6)where *C* represents the candidate gene set of some disease, and it contains all the genes with locations fall into the disease loci.

The weights *v_k_* of the graphlets in Equation (4) can be set by experience or machine learning from the datasets. In this part, linear regression was adopted to calculate the weights. When validating the performance of the algorithm, the disease dataset were divided into two parts: test dataset and training dataset. Training dataset was used to obtain the weights by regression and the test dataset was used to validate the algorithm.

Equation (4) was rewritten as
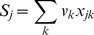
(7)
*x_jk_* was calculated by



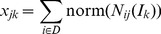
(8)When using training dataset, *s_j_* and *x_jk_* in the equation were known, and *v_k_* was unknown.

Then, the weight *v_k_* was calculated by the equation as following
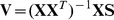
(9)

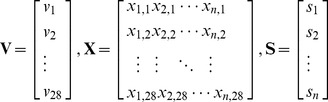
(10)
*s_j_* denotes whether the gene is a disease gene or not. When the *j*th gene is a disease gene, *s_j_*  = 1; when the *j*th gene is not a disease gene, *s_j_*  = 0.

### 2.4 Leave-one-out cross-validation

Leave-one-out cross-validation was applied to evaluate the performance of the graphlet interaction [14]. For every disease family, the genes were ranked according to the scores. When setting a threshold *S_0_*, the genes with scores less than *S_0_* were discarded. Meanwhile, locations of the genes were checked if they were contained within the interval known to be associated with the corresponding disease. The genes which both located in the disease loci and scored above the threshold *S_0_* were identified as positive genes.

For every disease, the algorithm was carried out for several times according to the number of the known disease genes. In each time, one of the disease genes was left out as unknown. If the gene left out by the algorithm was identified as a positive gene described above, it was a true positive (*TP*). The false positives (*FP*) were the positive genes described above which were not the known disease gene. The false negatives (*FN*) were the genes which were disease genes left out by the algorithm and were not identified as the positive genes described above. The true negatives (*TN*) were the genes which were not the known disease genes and were not identified as the positive genes. After obtaining the scores of all genes, the threshold was altered from the highest score to the lowest score. *TP*, *FP, TN* and *FN* were calculated corresponding to every threshold value.

The precision-recall (*P-R*) curves were plotted to show the performance of different algorithm in different conditions. The precision was calculated by *TP*/(*TP*+*FP*) and the recall was calculated by *TP*/(*TP*+*FN*). The whole performance also was represented by the maximum *F*-scores which were calculated by *F  = 2pr*/(*p+r*). Receiver operating characteristic (*ROC*) curves were also used to show the performance. The horizontal coordinate of *ROC* curves was false-positive-rate (*FPR*) which was calculated by *FPR*  =  *FP*/(*FP* + *TN*) and the longitudinal coordinate was true-positive-rate (*TPR*) which was calculated by *TPR*  =  *TP*/(*TP* + *FN*).

### 2.5 Data Sources

The human disorders and corresponding disease genes came from OMIM database [2] which focuses on the relationship between phenotype and genotype and updates daily. The data contains 5662 disease genes. There are 3871 unique disease genes because some genes are duplicated and participated in different diseases. The semantic similarities of the diseases were calculated to determine the disease families. The diseases with similarity values more than 0.3 were considered to be one disease family. All the diseases were grouped into 1871 disease families. Some disease families only had one disease gene, which could not be tested by leave-one-out cross validation. There were 876 disease families which having more than 2 disease genes.

To compare with random walk [18], Endeavour [19], and neighborhood based method [13], a data subset of disease genes was used. The previous published researches [18], [19] used the datasets which contained 783 and 627 disease genes, respectively. Hence a data subset contained 42 disease families which were random picked. It contained 741 distinct genes, which was similar to the above two approaches. The data subset included diseases with disease genes from 3 (Pulmonary hypertension) to 121 (deafness), and the average disease genes of one disease was 21.2 (Table S1).

When using the disease data subset to validate the performances of algorithms, the subset was used as test data. The data of the other diseases were used as training data to calculate the weights by liner regression. When using the whole dataset to validate the performances, the whole dataset was divided into 10 parts. Each part was used as test data, and the others were used as training data to calculate the weights. The disease genes reduplicated in the test and training data were deleted from the training data to the results believable.

OMIM also provides the location information of disease genes. There are 1591 different locations of the diseases. In NCBI human gene database, the genes located in these 1591 locations were used as candidate genes.

The interactome networks were integrated by PPIs and pathways. The PPIs came from Human Protein Reference Database (HPRD) [26], and Biological General Repository for Interaction Datasets (BioGRID) [27]. The pathways came from Kyoto Encyclopedia of Genes and Genomes (KEGG) database [28] and included two parts, i.e. metabolism pathways and non-metabolism pathways. The dataset from HPRD contains 9515 unique proteins and 36985 interactions. The dataset from BioGRID contains 7349 proteins and 21833 interactions. The pathways from KEGG were integrated into a pathway network by VisAnt [29] which contained 3694 proteins and 36298 interactions. Only the main components of networks were preserved to keep the network as a connected graph. All the networks were considered as undirected and unweighted. Then the above networks were integrated into a large network. The final integrated interactome network included 11696 nodes and 78327 interactions.

## Results and Discussion

### 3.1 Protocol of the graphlet interaction approach

**Figure 2:**
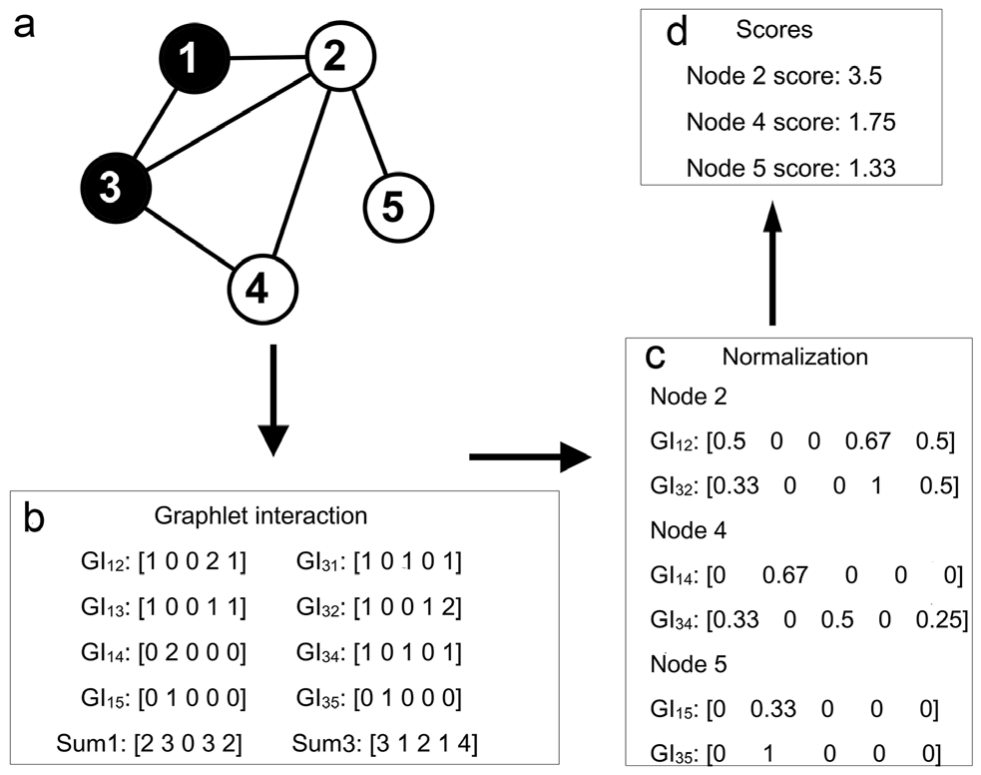
Protocol of disease gene identification using graphlet interaction. **a**. The small network was taken, and only 5 types of graphlet interaction isomers (*I_1_* to *I_5_*) were considered as an example. The black node 1 and node 3 were known disease genes. The protocol showed how to rank other genes according to known disease genes. **b**. The first step, calculation of the graphlet interaction between known disease gene (1, 3) and all the other genes. GI was the abbreviation of graphlet interaction, measured by a vector which had 5 elements corresponding to the numbers of the 5 types of graphlet interaction isomers (Figure. 1b *I_1_* to *I_5_*). The graphlet interactions from one disease gene were added. Sum1 and Sum3 were the summations of the graphlet interaction vectors from node 1 and node 3. **c**. The second step, normalization of the graphlet interaction. Every graphlet interaction was divided by the corresponding summation. GI_12_, GI_13_, GI_14_ and GI_15_ were divided by Sum1, and GI_31_, GI_32_, GI_34_ and GI_35_ were divided by Sum3. **d**. The third step, the graphlet interactions from the disease gene to every candidate gene were summated. Then, the elements of the summation were multiplied by the weights, and then added. The score of the node was obtained. For example, to get score of node 2, the normalized GI_12_ and GI_32_ were added and the summation vector [0.83 0 0 1.67 1] was obtained. The score was 0.83+0+0+1.67+1 = 3.5 (the weight of every element was 1 here).

Between every nodes pair, the number of different isomers was counted and the graphlet interaction was indicated by the vector of which the elements represented the number of the corresponding graphlet interaction isomer. When identifying disease genes, the score of every gene was calculated according to their graphlet interaction. Figure 2 uses 3-node graphlet as an example (Figure 2a) to show the protocol. In step one (Figure 2b), the graphlet interaction isomers from disease genes to all the other genes were counted by Equation (2) and Equation (3). The summation of the graphlet interaction vectors from every disease gene was computed by equation (6). In step two (Figure 2c), the graphlet interaction from every disease gene to every candidate gene was normalized by Equation (5). In step three (Figure 2d), all the normalized graphlet interactions of every candidate gene were added, and the score of every candidate gene was calculated by weighted summation as shown in Equation (4).

### 3.2 Graphlet interaction between disease genes

In this part, network properties of all genes were analyzed by using graphlet signature and graphlet interaction, and the difference between disease and random genes were compared. Random genes were picked from all genes in the network as the background.

Firstly, the average graphlet signature of disease genes and random picked genes were calculated, respectively. The *k*th element of the average graphlet signature vector was calculated by the equation


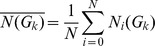
 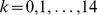 (11)

where *G_k_* represents the *k*th element of the graphlet signature vector, *N* *_i_*(*G_k_*) is the number of *G_k_* of the gene *i*, *N* is the total number of the genes.

**Figure 3:**
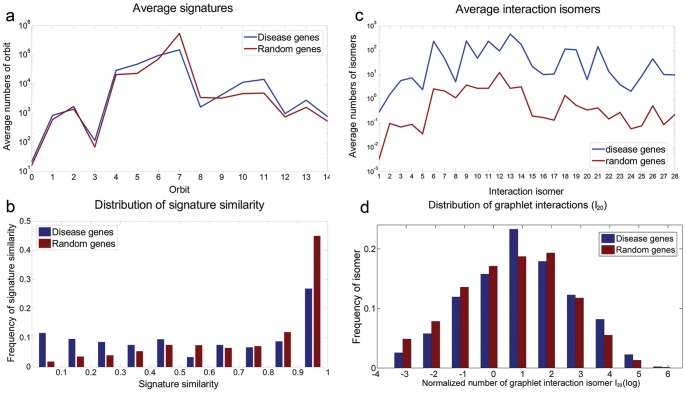
Compare disease genes with random genes using graphlet signature and graphlet interaction. Graphlet signature and graphlet interaction were applied and compared to distinguish the disease genes and random picked genes. **a**. The average signatures of disease genes (blue line) and random genes (red line); **b**. The distribution of graphlet signature similarities between disease genes (blue bars) and between random genes (red bars). The horizontal axis which was discretized to 10 grids represented the similarity from 0 to 1 and the longitude axis was the number of the gene pairs with corresponding similarities; **c**. The average number of graphlet interaction isomers between disease gene pairs (blue line) and random gene pairs (red line); **d**. The distribution of average graphlet interaction isomer *I_20_* of disease gene (blue bars) and random genes (red bars). The horizontal axis was the logarithmic number of the isomer, and the longitude axis was the normalized number of genes which had corresponding number of isomers.

Figure 3a shows the average graphlet signatures of disease genes and random genes in logarithmic scale. The correlation between the average graphlet signatures of disease genes and random genes was 0.9190, which suggested that the two averaged graphlet signatures were similar. *T*-test was used to analyze the difference between the two average signature and the *p*-value was 0.278, larger than the threshold 0.05. These results suggested the disease genes could not be identified from the random genes by the graphlet signature.

Secondly, the graphlet signature similarities were calculated between every gene pairs to distinguish the disease genes and random genes. The signature similarity was represented by the absolute value of the Pearson correlation coefficient of the graphlet signatures. The Pearson correlation coefficient was calculated as following
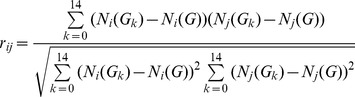
(12)Where *N* *_i_*(*G_k_*) is the *k*th element of the graphlet signature vector of the gene *i*, *N* *_i_*(*G*) is the average of all the elements of the graphlet signature vector of gene *i*.

The distributions of the graphlet signature similarities are shown in Figure 3b. The number of disease gene pairs with low similarity (≤ 0.5) was larger than random gene pairs, while the number of disease gene pairs with high similarity (> 0.5) was smaller than random gene pairs. It demonstrated that the similarity of graphlet signature did not distinguish disease genes from the background. Milenkovic, et al. applied graphlet similarity to identified cancer genes. However, the performance was not outstanding, and the max *F*-score of the method was less than 0.25 when using KNN clustering method [24], which also suggested that the graphlet signature might be not suitable to identify disease genes.

Thirdly, the average graphlet interactions of the disease gene pairs and the random gene pairs were investigated. The average number of the *k*th graphlet interaction isomer was calculated by equation
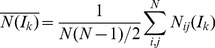
(13)Where *I_k_* is the *k*th graphlet interaction isomer and *N* *_ij_*(*I_k_*) is the number of the *I_k_* from gene *i* to gene *j*. *N* is total number of the genes to be calculated.

Figure 3c shows the curves which revealed the average graphlet interactions of disease gene pairs and random gene pairs. The average numbers of graphlet interaction isomers of disease gene pairs were much larger than random gene pairs. *T*-test was also used to evaluate the difference between the two average graphlet interactions and the *p*-value was 5.96×10^−11^. It suggested that the graphlet interaction was a feature to distinguish the disease genes from the background.

Finally, the graphlet interactions distributions of 28 types of isomers were investigated. The normalized numbers of graphlet interaction isomers of every disease gene and every random gene were calculated by the equation
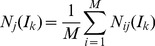
(14)where *N* *_ij_*(*I_k_*) is the number of the *k*th graphlet interaction isomer, *N* *_ij_*(*I_k_*) is the average number of graphlet interaction isomer from disease genes to the gene *j*, and *M* is the total number of disease genes.

Figure 3d shows the distribution of isomer *I_20_* as an example. The average numbers of *I_20_* from disease genes to all genes were from 0.027 to 532.3. The logarithm of average numbers was calculated to make the histogram clear and the values were from −3.6 to 6.277. The result showed that the numbers of random genes were more than disease genes when the logarithm were equal to or less than 0, while the most numbers of disease genes were more than random genes when the logarithm were more than 0. It meant that the disease gene pairs had larger numbers of graphlet interaction isomers than the disease-random gene pairs. The distributions of all the normalized number of graphlet interaction isomers are shown as Figure S1.

The above results revealed that the graphlet interaction between disease genes was different from random picked genes, but the graphlet signature was not. It suggested that graphlet interaction may be a better tool to identify disease genes. Therefore, the approach based on the graphlet interaction was designed and performed to identify the disease genes in the following part.

### 3.3 Performance of graphlet interaction in disease gene identification

The previous results suggested that a gene which had more graphlet interaction isomers with known disease genes had higher probability to be a disease gene as well. Hence, the new designed score was calculated based on graphlet interaction. To investigate whether the score can separate the disease genes from the background, the score distributions of disease genes and random genes were plotted (Figure S2). All the weights of the graphlet interaction isomers were set 1, here. Figure S2 shows that the scores of most disease genes were higher than random genes. The tendency was similar to the distribution of the graphlet isomers. The correlations between the graphlet interaction scores and the number of graphlet interaction isomers are shown as Figure S3.

To evaluate the precision of graphlet interaction on disease genes identification, the leave-one-out cross-validation was adopted and the *P-R* curves of graphlet interaction algorithm compared with previous approaches, i.e. random walk [18], Endeavour [19] and neighborhood based method [13], [14], were evaluated.

**Figure 4:**
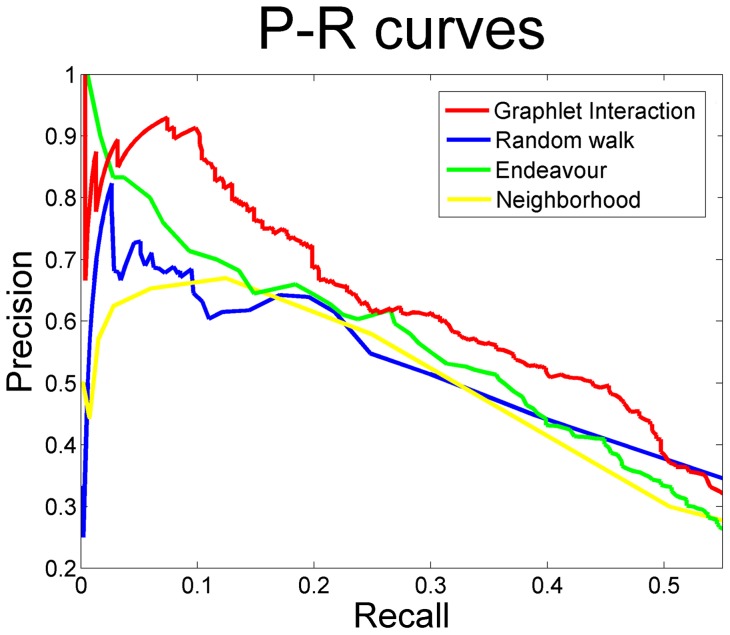
Performance of the graphlet interaction comparing with random walk, Endeavour and neighborhood based method. *P-R* curves of graphlet interaction approach (red line), random walk (blue line), Endeavour (green line) and neighbourhood based method (yellow line) in identifying disease genes. The graphlet interaction approach obtained the highest precision in most areas.

Figure 4a shows the *P-R* curves of the four approaches, i.e. graphlet interaction, random walk, Endeavour and neighborhood when using the data subset. The graphlet interaction obtained higher precision in almost all range. The precision of the graphlet interaction obtained the maximum 100% at the small recall and more than 90% at the recall 10%, which were much higher than the other three approaches. As the recall increased, the precision of graphlet interaction was still higher than the other three approaches. It suggested that graphlet interaction performed better in predicting new disease related genes. The precision of Endeavour also obtained 100% at the small recall, but decreased rapidly and only obtained about 70% when the recall was 10%. The highest precision of random walk was 82.35%, at the recall 2.65%, and the precision decreased to 64.56% at the recall 10%. The neighborhood based method was chosen as a baseline approach. It considered a candidate gene as a disease gene if there were at least 1 linkage between the candidate gene and disease genes. Several points were obtained by increasing the number of linkages and the *P-R* curve of the neighborhood based method were plotted. The highest precision of neighborhood based method was 67%, when at least 3 linkages with disease genes were considered, at the corresponding recall 12.43%. The maximum *F*-score of the graphlet interaction based approach was 0.466 while the random walk was 0.415, Endeavour was 0.436 and neighborhood based method was 0.4227.

*ROC* curves shows the performance of graphlet interaction as well in Figure S4. The area below graphlet interaction curve is larger than the other three approaches. The statistic data (*TP*, *FP*, *TN* and *FN*) of the 4 approaches is in Table S2. The top 100 identified disease genes by the graphlet interaction are listed in Table S3.

Graphlet interaction performed better than Endeavour. It is probably because that Endeavour exploits different data sources just by statistics, but does not consider the complex relationships between the genes in the network. Random walk algorithm considers the effect of network structure on the gene relationship. However, in the process of the “random walk”, the scores between two genes are mainly determined by the direct connection. If a gene has indirect link to a known disease gene, it is hardly identified. The neighborhood based method also just considers the direct connection between a candidate gene and disease genes.

The graphlet interaction approach considers not only the direct but also the indirect connections. The graphlet interaction includes 28 different types of linkages. There are 8 isomers which contain node pairs which does not link each other directly in the graphlet interaction. The score of a gene will be high enough if it connects with known disease genes by many graphlet, even though there is no direct connection between them.

Also, the graphlet interaction isomers reflect different topological structures. Every graphlet interaction isomer represents a unique topological structure, and they are non-isomorphic. The graphlet interaction approach tends to identify genes with high degree to be disease genes. Figure S5a shows a small network and just graphlet with not more than 3 nodes were calculated as an example. In the small network, node A is a known disease gene. Node B and node C are candidate genes. Using both neighborhood based method and random walk, node B and node C have the same relationship with A. However, the graphlet interactions of them are quite different. The graphlet interaction vector between A and B is [1 0 1 3 0] and between A and C is [1 0 1 0 0]. The score of node B is 2.0 while the score of node C was 1.0. Genes with higher degree tend to play more important roles in biological function. The property of graphlet interaction made it perform better than other approaches.

The graphlet interaction tends to identify candidate genes which are in the same complex with disease genes as disease genes too. Figure S5b shows another example. In the small network, node A is disease gene. Node B and C are candidate genes. Node C is the neighbor of A, but B is not. Using neighborhood based method, node C is more likely to be a disease gene than node B. However, A and B are in the same complex, but C is not. The graphlet interaction score of B is 1.0, which is higher than that of C (0.75). Genes in the same complex often participate in the same function, and the graphlet interaction approach tends to identify genes in the same complex with disease genes.

**Figure 5:**
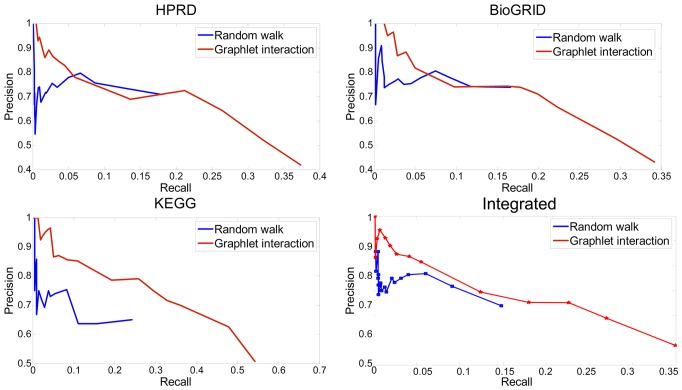
Performance of graphlet interaction and random walk using different networks. *P-R* curves of graphlet interaction approach and random walk using different data sources. **a**, HPRD network; **b**, BioGRID network; **c**, KEGG network; **d**, the integrated network.

To avoid bias, the whole disease datasets were used to validate the performance of graphlet interaction approach compared with random walk by leave-one-out cross-validation. Random walk was the widely used approach based on interactome network as graphlet interaction. 3 networks constructed from different data sources, i.e. HPRD, BioGRID and KEGG, and the integrated network were used respectively. The graphlet interaction performed better than the random walk (Figure 5). Among the 4 networks, graphlet interaction performed the best and obtained higher precision at all recall value by using KEGG network.

### 3.4 Predicting new disease gene

**Figure 6:**
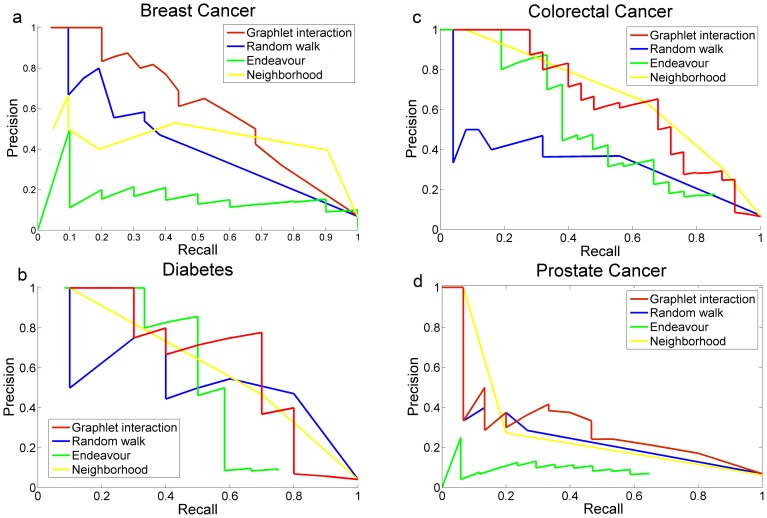
Performance in disease genes identification of 4 common diseases. *P-R* curves of graphlet interaction approach, random walk and Endeavour in disease gene identification of four common diseases. **a**, Breast cancer; **b**, Colorectal cancer; **c**, Diabetes; **d**, Prostate cancer.

Furthermore, to demonstrate the ability of the graphlet interaction to identify disease genes, new disease genes of the 4 common diseases, i.e. breast cancer, colorectal cancer, diabetes and prostate cancer, were identified by the approach. The performance of the graphlet interaction approach was compared with random walk and Endeavor, respectively. The *P-R* curves showed the precision of every disease (Figure 6). It revealed that graphlet interaction approach performed better than the other two methods. The performances of graphlet interaction approach and random walk were more stable than Endeavour, which obtained the highest precision at the largest recall of diabetes but very low precision of breast cancer and prostate cancer. The reason may be that in the annotations of KEGG, the disease genes of colorectal cancer and diabetes were included, but not the breast cancer and prostate cancer.

Then, new disease genes of the 4 diseases were identified by graphlet interaction approach. The genes were ranked according to the graphlet interaction scores. Genes which were not included in the OMIM database were considered as new disease genes if they were ranked ahead. Table 1 lists the top 20 ranked genes of the 4 diseases. 34 genes among the total 80 genes being in the OMIM database and the other 46 genes were new disease genes identified by our method. These new identified genes were checked whether some other researchers had identified them as disease genes. 31 genes among the 46 new identified disease genes were reported to be related to the corresponding diseases in the literatures and labeled by “#” in Table 1. For example, PIK3R5 was found to be related to both breast cancer and colorectal cancer verified by Wood, et al [30]. CDH3 [31], PRKDC [32] and PRKCI [33] had all been reported to be the breast cancer related genes. RHOA was identified by Wever, et al [34] as a colorectal cancer related gene. HRAS [35], VAV1 [36], PDE3B [37] and TYK2 [38] were suggested to relate to diabetes. CDH3 was also a prostate cancer related gene besides breast cancer [39]. CASP7 [40], SMC3 [41], PTPN12 [42] and GTF2I [43] were validated as prostate cancer related genes by different researchers and various experiments. The above researches further verified our identifications, and suggested that the approach based on graphlet interaction could obtain high precision in identifying disease genes. There was no apparent evidence to prove the relationship between the other 15 new identified genes and the corresponding disease, for example MOS, SOS1, CTSD and YWHAG. Our results suggested that these new identified genes have high probability to be the disease genes.

**Table 1:** Disease gene identification of 4 common diseases.

Rank	Breast cancer		Colorectal cancer		Diabetes		Prostate cancer	
	Genes	Scores	Genes	Scores	Genes	Scores	Genes	Scores
1	TP53	19.53	TP53	16.06	INS	10.65	AR	9.951
2	ESR1	8.620	MLH1	15.51	INSR	6.524	CDH3* #	6.052
3	PIK3R5* #	8.503	APC	13.87	HRAS* #	5.376	CASP7* #	5.557
4	AKT1	7.455	CTNNB1	13.76	HNF1A	5.277	YWHAG*	5.298
5	PIK3CA	6.457	EP300	12.07	HNF1B	5.198	PTEN	4.057
6	CDH3* #	6.045	RHOA* #	8.696	KCNJ11	4.187	CDH1	2.267
7	PRKDC* #	5.044	AKT1	8.151	ABCC8	4.187	SMC3* #	2.140
8	KRAS	3.118	NRAS	7.499	VAV1* #	4.075	ASCC2*	2.104
9	PRKCI* #	2.213	PIK3R5* #	7.275	PDE3B* #	4.039	PTPN12* #	0.858
10	TSG101	1.501	PMS2	6.977	TYK2* #	2.906	GTF2I* #	0.826
11	CHD3* #	1.467	MSH2	6.646	CDC37* #	2.608	ACTB* #	0.826
12	KHDRBS1*	1.452	MSH6	6.344	SMARCA4* #	1.865	LPL* #	0.738
13	SMURF2* #	1.408	DVL2* #	5.886	GCK	1.472	RAC1* #	0.639
14	MTA1* #	1.331	PIK3CA	5.726	CTSD*	1.101	SF3B3*	0.627
15	DVL2* #	1.236	RAC1* #	5.486	GRB7* #	1.000	XPO1*	0.618
16	EPS15* #	1.229	SOS1*	5.419	PIP4K2B*	0.911	DGKZ*	0.604
17	MOS*	1.110	PRKCE*	4.433	DNM2*	0.870	LIPF*	0.586
18	POLR2A* #	1.076	PFAS*	4.041	AKT2	0.703	RNASEL	0.569
19	CDH1	0.953	BUB1	3.017	BCL3* #	0.699	CCAR2	0.527
20	TAB2* #	0.904	PMS1	2.812	RAB3D*	0.652	BRCA2	0.527

* means the genes were not included in the disease gene list of OMIM.

# means the genes which not in OMIM were verified by literatures.

## Conclusion

We presented a new approach which identified disease genes based on interactome network. The approach applied graphlet interaction to determine whether a gene had closely relationship with known disease genes. The scores of the graphlet interactions between candidate genes and known disease genes were calculated and genes were ranked according to the scores. A gene with higher scores had higher probability to be a new disease gene. The performance of the approach was evaluated by leave-one-out cross-validation, and compared with random walk, Endeavour and neighborhood based method. The results showed that the approach based on graphlet interaction perform better than the other methods. To avoid bias, the approach was carried out on 3 independent networks and the integrated network, and the results showed the similar tendency. Finally, the approach was applied to identify new disease genes of 4 common diseases, and proved that these identified new disease genes had high probability to be disease genes.

## Supporting Information

Figure S1 **Normalized number distribution of graphlet interaction isomers I_1_ to I_28_.** **a.** Equal numbers of disease genes and random genes were chosen and the normalized number distributions of graphlet interaction isomers were compared. Because there were too many zeros values, the bars of zero values were not shown to make the histogram readable. The horizontal axis is the normalized number of isomers. The longitude axis is the number of genes corresponding to the normalized number of isomers. **b.** Equal numbers of disease genes and random genes which have non-zeros values were chosen and the normalized number distributions of graphlet interaction isomers were compared. The horizontal axis is the normalized number of isomers, which was logarithmic scaled to make the histogram clear. The longitude axis is the corresponding number of genes.
(TIF)

Figure S2 **Distribution of graphlet interaction scores, comparing between disease genes and random genes.** Equal numbers of disease genes and random genes were chosen and the distributions of the graphlet interaction scores were compared. Because there were too many zero values, the bars of zero values were not shown to make the histogram readable.
(TIF)

Figure S3 **Correlations of graphlet interaction scores and numbers of graphlet isomer.** In the figures, every point meant a gene. The horizontal coordinate meant the logarithmic graphlet interaction score, and the longitudinal coordinate meant the logarithmic average number of graphlet interaction isomer.
(TIF)

Figure S4 ***ROC*** **curves of graphlet interaction approach (red line), random walk (blue line), Endeavour (green line) and neighbourhood based method (yellow line).** The horizontal coordinate meant the false-positive-rate and the longitudinal coordinate meant the true-positive-rate. The graphlet interaction approach curve was above others in most region. It meant that when getting the same false positive, graphlet interaction obtained higher true positive.
(TIF)

Figure S5 **Schema models to reveal the advantages of graphlet interaction.** **a.** A is known disease gene. B and C were candidate genes. B had high degree; **b.** A was known disease gene. B and C were candidate genes. B was in the same complex with A.
(TIF)

Table S1 **Data subset of disease genes which include 904 disease genes and 42 disease families.**
(XLSX)

Table S2 ***TP*** **,** ***FP*** **,** ***TN*** **,** ***FN*** **and corresponding scores of graphlet interaction, random walk, Endeavour and neighborhood based method.**
(XLSX)

Table S3 **Top 100 candidate genes ranked by graphlet interaction scores.**
(XLSX)
