# Supplementary figures and images for: Identification of Human Disease Genes from Interactome Network Using Graphlet Interaction
Source: PLoS One. 2014 Jan 22;9(1):e86142. doi: 10.1371/journal.pone.0086142 (PMC3899204; doi:10.1371/journal.pone.0086142)

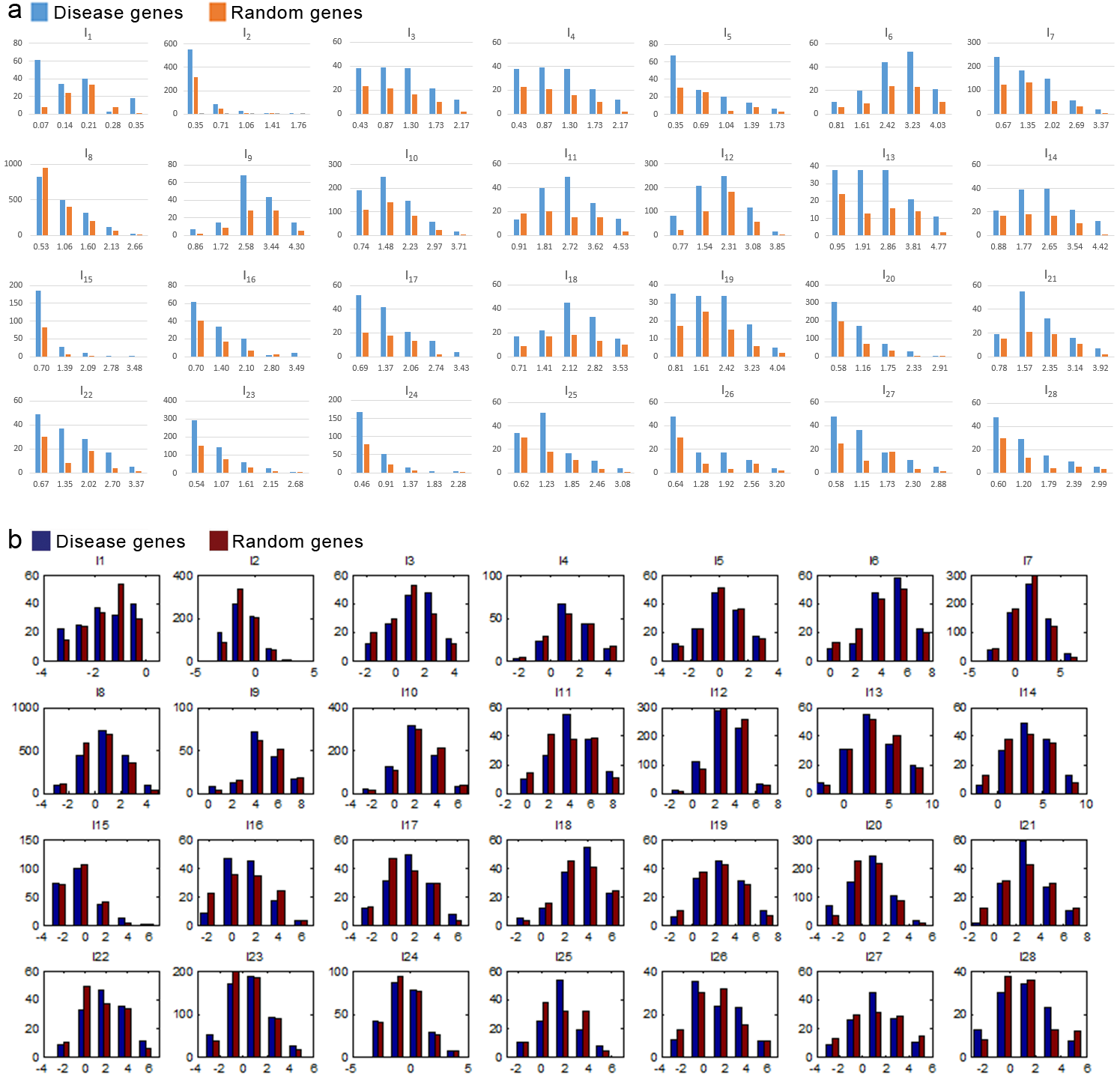

Supplement: Figure S1 — Normalized number distribution of graphlet interaction isomers I1 to I28. a. Equal numbers of disease genes and random genes were chosen and the normalized number distributions of graphlet interaction isomers were compared. Because there were too many zeros values, the bars of zero values were not shown to make the histogram readable. The horizontal axis is the normalized number of isomers. The longitude axis is the number of genes corresponding to the normalized number of isomers. b. Equal numbers of disease genes and random genes which have non-zeros values were chosen and the normalized number distributions of graphlet interaction isomers were compared. The horizontal axis is the normalized number of isomers, which was logarithmic scaled to make the histogram clear. The longitude axis is the corresponding number of genes. (TIF) [file pone.0086142.s001.tif]

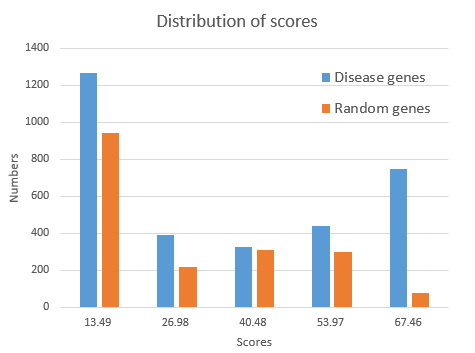

Supplement: Figure S2 — Distribution of graphlet interaction scores, comparing between disease genes and random genes. Equal numbers of disease genes and random genes were chosen and the distributions of the graphlet interaction scores were compared. Because there were too many zero values, the bars of zero values were not shown to make the histogram readable. (TIF) [file pone.0086142.s002.tif]

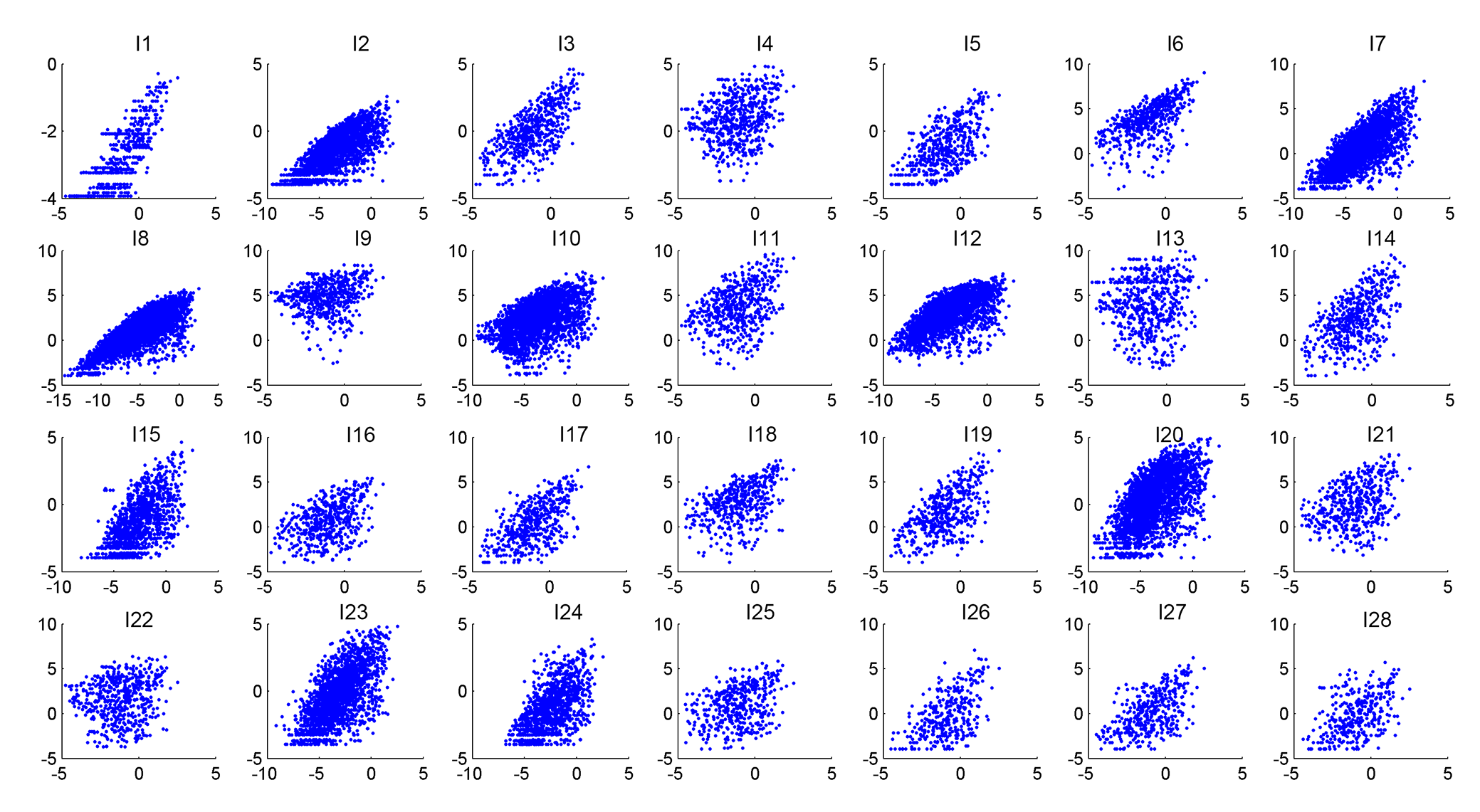

Supplement: Figure S3 — Correlations of graphlet interaction scores and numbers of graphlet isomer. In the figures, every point meant a gene. The horizontal coordinate meant the logarithmic graphlet interaction score, and the longitudinal coordinate meant the logarithmic average number of graphlet interaction isomer. (TIF) [file pone.0086142.s003.tif]

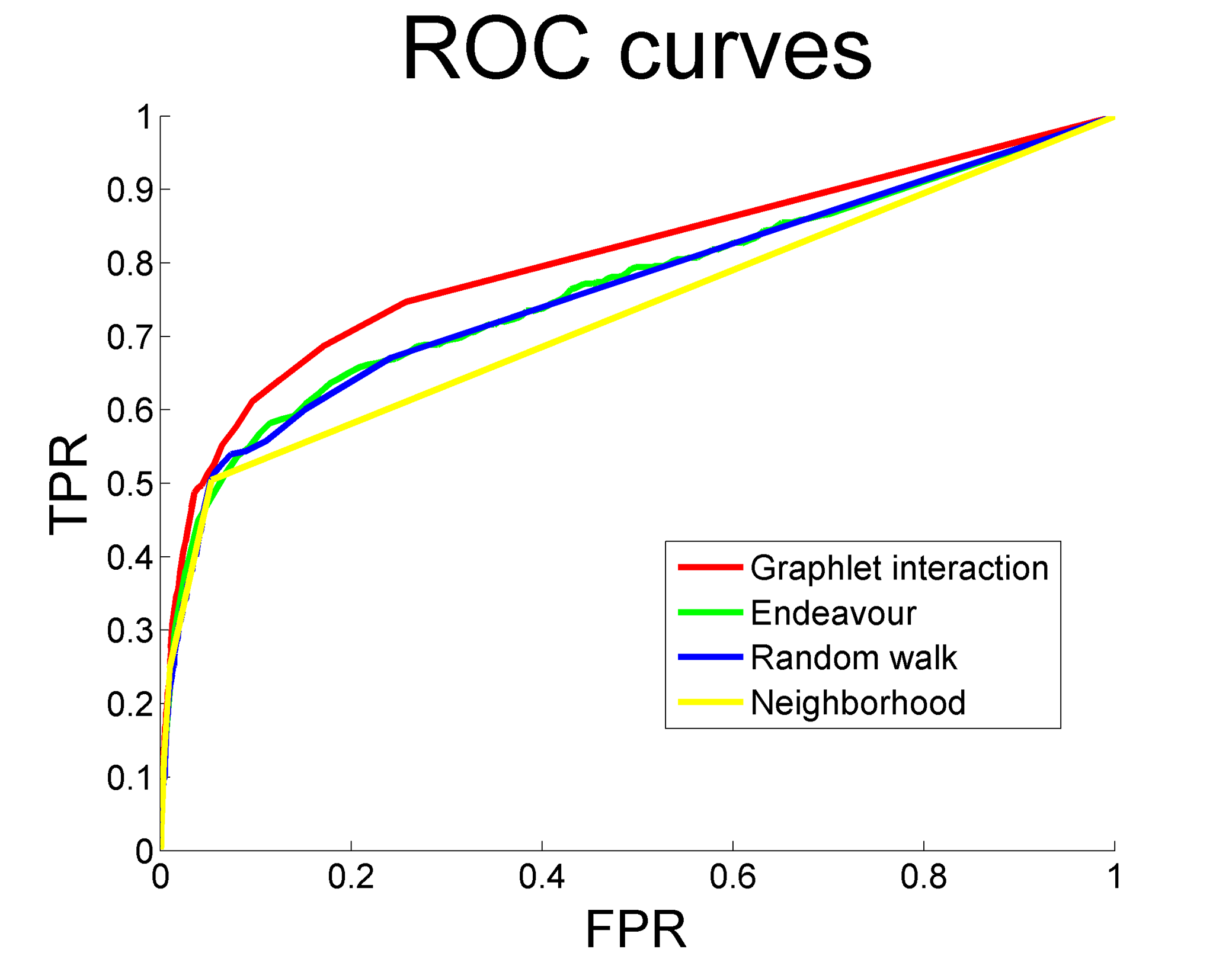

Supplement: Figure S4 — ROC curves of graphlet interaction approach (red line), random walk (blue line), Endeavour (green line) and neighbourhood based method (yellow line). The horizontal coordinate meant the false-positive-rate and the longitudinal coordinate meant the true-positive-rate. The graphlet interaction approach curve was above others in most region. It meant that when getting the same false positive, graphlet interaction obtained higher true positive. (TIF) [file pone.0086142.s004.tif]

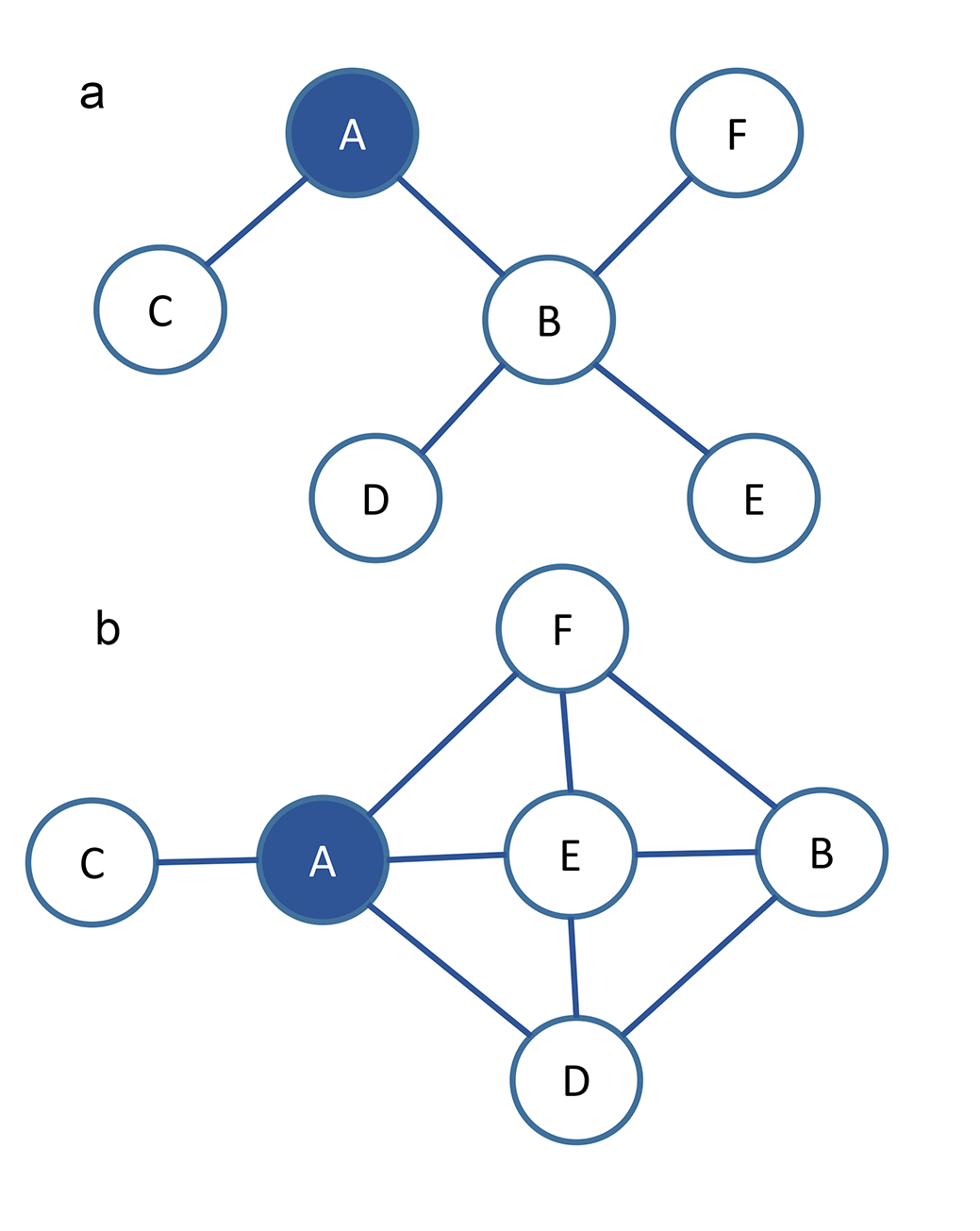

Supplement: Figure S5 — Schema models to reveal the advantages of graphlet interaction. a. A is known disease gene. B and C were candidate genes. B had high degree; b. A was known disease gene. B and C were candidate genes. B was in the same complex with A. (TIF) [file pone.0086142.s005.tif]
